# GPCR-mediated PI3K pathway mutations in pediatric and adult thyroid cancer

**DOI:** 10.18632/oncotarget.26993

**Published:** 2019-06-25

**Authors:** Avaniyapuram Kannan Murugan, Ebtesam Qasem, Hindi Al-Hindi, Ali S. Alzahrani

**Affiliations:** ^1^ Division of Molecular Endocrinology, Department of Molecular Oncology, Riyadh 11211, Saudi Arabia; ^2^ Department of Pathology and Laboratory Medicine, Riyadh 11211, Saudi Arabia; ^3^ Department of Medicine, King Faisal Specialist Hospital and Research Centre, Riyadh 11211, Saudi Arabia

**Keywords:** thyroid cancer, mutations, PTEN, oncogene, PIK3CA

## Abstract

Whole exome sequencing (WES) recently identified frequent mutations in the genes of GPCR-mediated PI3K pathway (*LPAR4*, *PIK3CA*, and *PTEN*) in a Chinese population with papillary thyroid cancers (PTCs). The study found *LPAR4* mutations as novel gene mutations in adult population with differentiated thyroid cancer (DTC). Here, we determine the prevalence of somatic mutations in this pathway (*LPAR4* (exon 1), *PIK3CA* (exons 9 and 20) and *PTEN* (exons 5, 6, 7 and 8) in 323 thyroid samples consisting of 17 multinodular goiters (MNG), 89 pediatric DTCs, 204 adult DTCs, and 13 aggressive thyroid cancers including 10 poorly differentiated (PDTC) and 3 anaplastic thyroid cancer (ATC) from another ethnic population. We found 3.37% and 2.45% (includes Q214H, a novel *PTEN* mutation) in GPCR-mediated PI3K pathway of pediatric and adult DTCs, respectively. Analyses of 507 DTCs from thyroid Cancer Genome Atlas data (TCGA) revealed a low prevalence of mutations in this pathway (1.18%). In 13 cases with PDTC and ATC, we found no mutation in genes of this pathway. By contrast, analyses of 117 aggressive thyroid cancers (PDTC and ATC) from TCGA showed 13% of mutations in this pathway. Moreover, analyses of 1080 pan-cancer cell lines and 9020 solid tumors of TCGA data revealed high rates of mutations in this pathway (cell lines, 24.8%; tumors, 24.8%). In addition, *PIK3CA* + *PTEN* (*p* = <0.001) and *LPAR4* + *PIK3CA* (*p* = 0.003) significantly co-occurred. Our study reveals a low prevalence of GPCR-mediated PI3K pathway mutations both in pediatric and adult DTCs corroborating the TCGA data and suggests a significant role of this pathway only in a small portion of DTCs. The high prevalence of mutations in this pathway in other solid malignancies suggests an important role in their pathogenesis making it an attractive target for therapeutic intervention both in a small subset of DTCs and other solid cancers.

## INTRODUCTION

Thyroid cancer is the most common endocrine malignancy with complex carcinogenesis mechanisms. The incidence of thyroid cancer increased over the past 4 decades [1, 2]. Thyroid cancer is the second most common cancer in women next to breast cancer and the fifth most common cancer in all gender in Saudi Arabia [3]. Differentiated thyroid cancer (DTC) accounts for ~90% of all thyroid cancers and is comprised of papillary thyroid cancer (PTC) and follicular thyroid cancer (FTC) [4].

Major advances in DNA sequencing technologies revealed various molecular abnormalities in coding [4, 5] and non-coding genes [6] of several signaling pathways in thyroid cancer. Somatic genetic alterations in these pathways are mostly mutually exclusive in the course of malignant transformation with varying rates among different populations. DTCs frequently (~70%) harbor activating somatic alterations in the genes of mitogen-activated protein kinase (MAPK) signaling pathway [7, 8]. The most prevalent genetic alterations are the hotspot point mutation *BRAF^V600E^* [9, 10], *RAS* single point mutations, and fusions in *RET/PTC* [11], *NTRK1/3* and *PAX8-PPARγ* [8, 12]. A low frequency of mutations has also been reported in the receptor tyrosine kinases (RTKs) including *EGFR* [13] and members of the phosphatidylinositol-3 kinase (PI3K) pathway such as *PIK3CA*, *PTEN* and *AKT1* [14–16]. Previous studies also reported some point mutations in the genes of metabolic and regulatory pathways which include *IDH1* [17] and *TERT*. Somatic alterations of these genes are strongly associated with distinct clinicopathological features of the tumors [18, 19]. Next-generation whole genome sequencing (WGS) of PTCs revealed additional somatic driver alterations that include *EIF1AX*, *PPM1D*, and *CHEK2* [4]. Although similar histology is observed in both pediatric and adult cases, the genetic profile and the frequency of the commonly identified genetic mutations in adult DTCs are different from those in the pediatric DTCs [20] suggesting significant differences in the pathogenesis between adult and pediatric DTCs. Poorly differentiated thyroid cancer (PDTC) and anaplastic thyroid cancers (ATC) are aggressive subtypes of thyroid cancer [7, 8]. In addition to the frequent occurrence of the oncogenic mutations of *BRAF^V600E^* and *NRAS*, mutations in the other critical genes such as *TERT*, *TP53*, *EIF1AX*, *PIK3CA*, *PTEN*, *ALK*, and *mTOR* mutations were frequently detected in PDTC and ATC [5, 7, 8, 21, 22, 23].

Lysophosphatidic acid receptor 4 (LPAR4) is a 41.895 kDa protein molecule encoded by 370 amino acids (except stop codon) which are translated from 1110 nucleotides. This gene is located on the X chromosome (Xq21.1). LPAR4 is also variably called as G-protein coupled receptor 23 (GPCR23), purinergic receptor 9, P2Y purinoceptor 9, P2Y5-like receptor, LPA receptor 4, and GPR23. It is one of the six subtypes (LPAR1-6) of G protein-coupled transmembrane receptors (GPCR) that is activated by ubiquitous bioactive ligand named lysophosphatidic acid (LPA), an extracellular biological lipid that is framed by a glycerol, a fatty acid, and a phosphate. LPARs are classified into two families, the endothelial cell differentiation gene (Edg) family (LPAR1-3) and the non-Edg purinergic receptor family (LPAR4-6) [24]. The LPAR4 has three vital functions at the molecular level which includes G protein-coupled receptor activity, binding to lysophosphatidic acid, and lysophosphatidic acid receptor activity. It is mainly involved in the signaling pathways of G protein-coupled receptor, positive regulation of cytosolic calcium ion concentration involved in phospholipase C-activating G protein-coupled signaling, and positive regulation of Rho protein signal. LPA induced LPAR-mediated PI3K/AKT signaling via Gαi/o subunits is implicated in various biological functions including proliferation, motility, invasion, and tumorigenicity and it confers chemotherapy-resistance in certain cancers [25].

Recently, whole exome sequencing of a Chinese population with DTCs identified frequent somatic mutations in the genes of the GPCR-mediated PI3K pathway [26]. However, to date, this axis was not analyzed in detail in other populations. Furthermore, the roles of this axis were not studied in pure pediatric and adult cases. Here, we studied mutations in the major genes (*LPAR4*, *PIK3CA,* and *PTEN*) of the GPCR-mediated PI3K pathway in a large series of pediatric and adult DTCs, and in aggressive thyroid cancer (PDTC and ATC) samples (*n =* 323) from a Saudi Arab population. We also analyzed the most comprehensive TCGA study data of DTCs (*n =* 507) and aggressive thyroid cancers (PDTCs and ATCs) (*n =* 117) to determine if there are differences in the rates of these mutations among different populations. Further, to understand the prevalence and importance of genetic mutations of the GPCR-mediated PI3K pathway in other types of human cancers, we comprehensively analyzed the largest cohort of TCGA data from pan-cancer cell lines (*n =* 1080) and pan-tumor samples (*n =* 9020). This study on thyroid cancer in the pediatric and adult population along with the detailed analyses of international data provides a comprehensive data on the GPCR-mediated PI3K pathway not only in pediatric, adult DTCs and aggressive thyroid cancers (PDTC and ATC) but also in other human solid tumors.

## RESULTS

### GPCR-mediated PI3K pathway genes (*LPAR4, PIK3CA,* and *PTEN*) are mutated both in pediatric and adult DTCs with similar low prevalence

**Figure 1:**
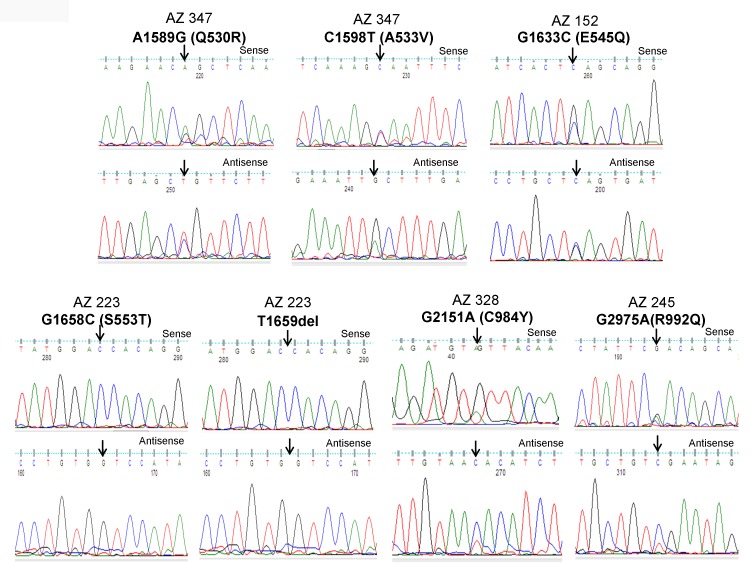
Identification of *PIK3CA* mutations in pediatric and adult DTCs. The illustration shows the sequencing electropherogram of the *PIK3CA* gene in various samples. The sequence shown on top and bottom indicates the sense and antisense strand, respectively. Altered nucleotide positions and its related amino acid (within bracket) are shown on top of each corresponding sequence. Mutated nucleotides are indicated by arrows. Nucleotide number refers to the position within the coding sequence of the *PIK3CA* gene, where nucleotide position 1 indicates the first nucleotide of the translation initiation codon. All samples were sequenced in 2 repeated experiments with independent PCR by sense and antisense primers.

Overall, GPCR-mediated PI3K pathway genes harbored mutations in 3.37% (3/89) and 2.45% (5/204) of pediatric and adult DTCs, respectively. *PIK3CA* mutations occurred in 2.24% (2/89) of pediatric DTC (both cases were conventional PTCs) and in 1.5% of (3/204) adult DTC [two in follicular variant PTC (FV-PTC) and one in tall cell variant (TPTC)]. *PTEN* mutations were present in1.1% (1/89) of pediatric conventional PTC and 1% (2/204) adult DTC (1 conventional PTC and 1 follicular variant PTC). The former one harbored a novel mutation (Q214H). Overall, in both pediatric and adult DTCs, *PIK3CA* mutations were found in 1.7% (5/293) and the *PTEN* mutations were found in 1% (3/293). We did not identify *LPAR4* gene mutations in any of the samples analyzed. MNG samples had no mutation in any of the genes analyzed. We also found no mutation in the *LPAR4*, *PIK3CA* and *PTEN* genes of the 13 cases of PDTCs and ATCs (aggressive thyroid cancers). All the mutation positive samples were further analyzed for hotspot and non-hotspot mutations of commonly mutated genes in DTC which include *BRAF^V600E^* and *TERT* promoter mutations. We found 2 samples concomitantly harboring *PIK3CA* and *BRAF* mutations and one of the two *BRAF* mutations was a non-hotspot frameshift mutation (1798inTAC) and the other one was the common *BRAF*^V600E^ mutation. Similarly, two samples with *PTEN* mutations coexisted with *BRAF*^V600E^ hotspot mutation. However, we did not find *TERT* promoter mutations in any of the sequenced samples (Figure 1 and 3, Tables 1 and 2).

**Table 1:** Summary of mutations identified in *LPAR4*, *PIK3CA* and *PTEN* genes of GPCR-mediated PI3K pathway in pediatric and adult thyroid cancer

Samples	Tumor subtype	Number of Samples	*LPAR4*	*PIK3CA*	*PTEN*
MNG		17	0/17 (0%)	0/17 (0%)	0/17 (0%)
Pediatric DTC	CPTC	72	0/72 (0%)	2/72 (2.7%)	1/72 (1.4%)
	FV-PTC	7	0/7 (0%)	0/7 (0%)	0/7 (0%)
	TPTC	2	0/2 (0%)	0/2 (0%)	0/2 (0%)
	DSC	3	0/3 (0%)	0/3 (0%)	0/3 (0%)
	FTC	3	0/3 (0%)	0/3 (0%)	0/3 (0%)
	HCC	2	0/2 (0%)	0/2 (0%)	0/2 (0%)
		**89**	**0/89 (0%)**	**2/89 (2.24%)**	**1/89 (1.1%)**
	**Overall**			**3/89 (3.37%)**	
Adult DTC	CPTC	114	0/114 (0%)	0/114 (0%)	1/114 (0.9%)
	FV-PTC	55	0/55 (0%)	2/55 (3.6%)	1/55 (1.8%)
	TPTC	29	0/29 (0%)	1/29 (3.5%)	0/29 (0%)
	DSC	3	0/3 (0%)	0/3 (0%)	0/3 (0%)
	HCC	3	0/3 (0%)	0/3 (0%)	0/3 (0%)
		**204**	**0/204 (0%)**	**3/204 (1.5%)**	**2/204 (1%)**
	**Overall**			**5/204 (2.45%)**	
	**Total DTCs**	**293**	**0/293 (0%)**	**5/293 (1.7%)**	3/293 (1%)
Aggressive cancer	PDTC	10	0/10 (0%)	0/10 (0%)	0/10 (0%)
	ATC	3	0/3 (0%)	0/3 (0%)	0/3 (0%)
		13	**0/13 (0%)**	**0/13 (0%)**	**0/13 (0%)**
				**0/13 (0%)**	

Abbreviations: CPTC, conventional papillary thyroid cancer; FV-PTC, follicular variant papillary thyroid cancer; TPTC, tall cell papillary thyroid cancer; FTC, follicular thyroid cancer; DSC, diffuse sclerosing type papillary thyroid cancer; HCC, Hürthle cell cancer; PDTC, poorly differentiated thyroid cancer; ATC, anaplastic thyroid cancer.

### Mutations are identified in important domains and predict potential function

**Figure 2:**
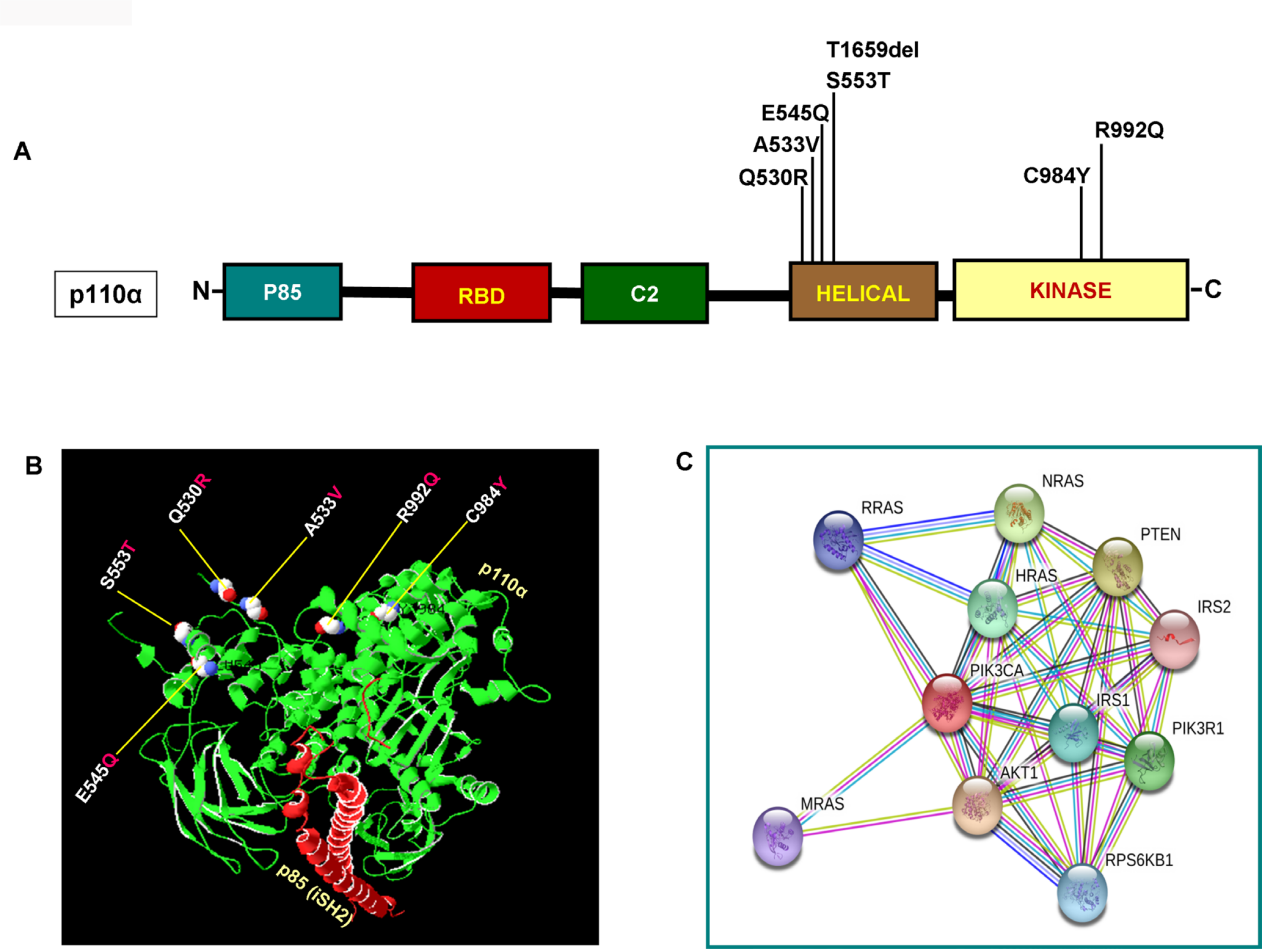
Functional domains of p110α and its interactome. (**A**) Schematic diagram of p110α protein. Shown are various somatic mutations identified in the *PIK3CA* gene of pediatric and adult DTCs and located in various corresponding domains of p110α. (**B**) Structure of p110α with DTCs associated mutations identified in this study. Shows ribbon diagram of native modeled structure of p110α /niSH2 (p85) as a heterodimer and its PDB ID is 2RDO. Mutated amino acid residues in DTCs were plotted in p110α native protein structure using SWISS PDB viewer. All the mutated residues are shown in a sphere shape, p110α is shown in green color and the niSH2 domain of p85α is shown in red color. (**C**) Interactome shows the interacting network of PIK3CA (p110α). The network nodes represent proteins and the red-colored node indicates the query protein and first shell of interactors. Bond in light blue and purple color shows known interactions from the curated database and experimentally determined, respectively. Bond in green, red and dark blue shows predicted interactions from gene neighborhood, gene-fusions, and gene co-occurrence, respectively. Black indicates co-expression while parrot green and pale blue shows text mining and protein homolog, respectively.

**Figure 3:**
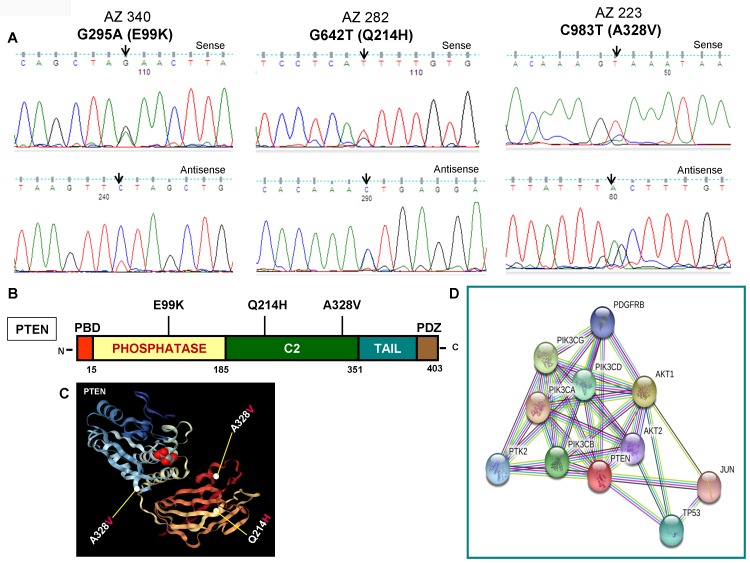
Identification of *PTEN* mutations, corresponding domains, and interactome of PTEN. (**A**) Chromatopherograms of the sequences of PTEN mutated samples. Sequences shown on the top and bottom indicate the sense and antisense strand, respectively of a sample. Altered nucleotide positions and its related amino acid (within bracket) are shown on top of each corresponding sequence. Arrows indicate the mutated nucleotides. Nucleotide number refers to the position within the coding nucleotides of the *PIK3CA* gene, where nucleotide position 1 shows the first nucleotide of the translation initiation codon. (**B**) Schematic diagram of PTEN protein. Marked-amino acids are various somatic mutations identified in the PTEN gene of pediatric and adult DTCs and located in various corresponding domains of PTEN. (**C**) A native modeled 3D structure of PTEN with pediatric and adult DTCs-associated mutations identified in this study. Ribbon diagram shows native modeled structure of PTEN protein and its PDB ID is 1D5R. Mutated amino acid residues in thyroid cancer are plotted in PTEN native protein structure using WebGL viewer as explained in materials and methods. All the mutated residues are marked in a sphere shape. (**D**) Interactome shows the interacting network of PTEN protein. The network nodes represent proteins and the red-colored node indicates the query protein and first shell of interactors. The bond represents protein-protein associations. Color of the bond and the type of associations are as mentioned above in Figure 2D.

As illustrated in Figure 2A and 2B, *PIK3CA* mutations occur in both exons 9 and 20 coding helical and kinase domains of the p110α, respectively. Five out of seven mutations accumulated in the helical domains while the other two were in the kinase domain of *PIK3CA*. Of the 5 helical domain mutations, only one mutation was found in the helical domain hotspot at codon E545 (E545Q) and other mutations were away from the helical domain hotspot. Six (Q530R, A533V, E545Q, S553T, C984Y, and R992Q) out of these seven *PIK3CA* mutations were missense single point mutations and one (T1659del) had a single nucleotide deletion (Table 2). To predict the interacting network of the *PIK3CA*, we used STRING network analysis tool and found that *PIK3CA* is an important key player in the signaling network as it closely interacts with other oncogenic drivers such as *PIK3R1*, *HRAS*, *NRAS*, *AKT*, etc (Figure 2C). These results suggest that the mutations are likely to be driver mutations in DTCs. Figure 3A–3C shows the *PTEN* mutations identified in DTCs and of 3 mutations, one occurred in the phosphatase domain, and other two in the C2 domain. To predict the PTEN interaction network, we constructed a PTEN interactome that revealed a strong interaction of PTEN with PIK3CA/B, AKT2/1, P53, PDGFR, etc. This result shows that PTEN is an important member of this network and mutations in the *PTEN* would likely impair its normal function suggesting that mutations identified are likely loss of function mutations (Figure 3D).

**Table 2:** Somatic mutations of GPCR-mediated PI3K pathway genes: *PIK3CA* and *PTEN* mutations identified in pediatric and adult DTCs

Tumor No	Group	Sex	Age	Histology	Gene	Exon	Nucleotide	Codon	Amino acid	Mutation	Status	*BRAF*	*TERT*	*COS*
AZ347	Pediatric	F	13	CPTC	*PIK3CA*	09	A1589G	CAG-CGG	Q530R	Missense	Heterozygous	-	-	+
AZ347	Pediatric	F	13	CPTC	*PIK3CA*	09	C1598T	GCA-GTA	A533V	Missense	Heterozygous	-	-	+
AZ152	Adult	F	44	FV-PTC	*PIK3CA*	09	G1633C	GAG-CAG	E545Q	Missense	Heterozygous	1798inTAC	-	+
AZ223	Adult	F	42	FV-PTC	*PIK3CA*	09	G1658C	AGT-ACT	S553T	Missense	Heterozygous	-	-	+
AZ223	Adult	F	42	FV-PTC	*PIK3CA*	09	T1659del	-	-	Frameshift	Homozygous	-	-	+
AZ328	Pediatric	F	13	CPTC	*PIK3CA*	20	G2951A	TGT-TAT	C984Y	Missense	Heterozygous	-	-	+
AZ245	Adult	F	62	TCPTC	*PIK3CA*	20	G2975C	CGA-CAA	R992Q	Missense	Heterozygous	V600E	-	+
AZ340	Pediatric	F	18	CPTC	*PTEN*	05	G295A	GAA-AAA	E99K	Missense	Heterozygous	V600E	-	+
AZ282	Adult	F	42	CPTC	*PTEN*	07	G642T	CAG-CAT	Q214H	Missense	Heterozygous	V600E	-	Novel
AZ223	Adult	F	42	FV-PTC	*PTEN*	08	C983T	GCA-GTA	A328V	Missense	Heterozygous	-	-	+

Abbreviations: M, male; F, female; FV-PTC, follicular variant papillary thyroid cancer; CPTC, conventional papillary thyroid cancer; -, no mutation; COS, Catalogue Of Somatic Mutations in Cancer; +, listed in the COSMIC database; Novel, novel mutation.

### Comprehensive TCGA data analyses of PTCs show a relatively low incidence of mutation in GPCR-mediated PI3K pathway genes

**Figure 4:**
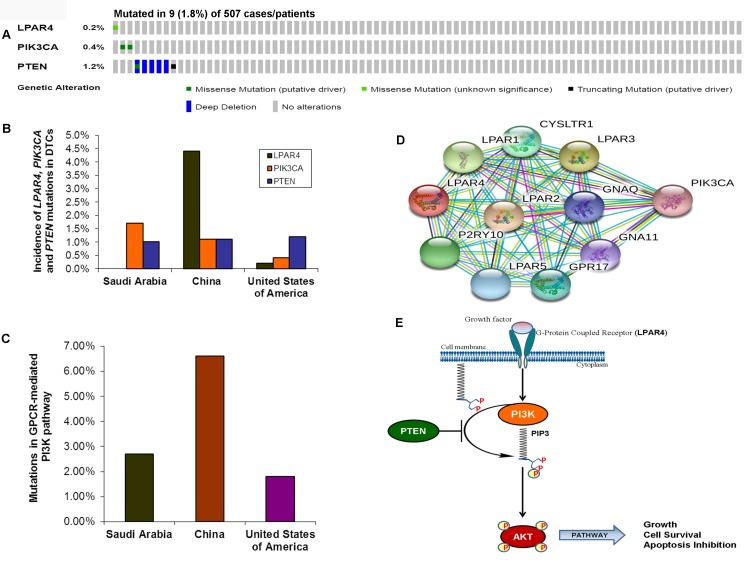
Prevalence of GPCR-mediated PI3K pathway mutations in DTCs. (**A**) OncoPrint of GPCR-mediated PI3K pathway- Thyroid cancer TCGA. The OncoPrint summarizes genomic alterations in *LPAR4, PIK3CA* and *PTEN* genes across the TCGA sample set. Each horizontal row indicates a gene and each vertical column shows a tumor sample. Green bars show nonsynonymous mutations and blue bars shows homozygous deletions. (**B**) Prevalence of LPAR4, PIK3CA, and PTEN in DTCs of various studies. The bar shows each *LPAR4, PIK3CA* and *PTEN* mutations in Sanger sequencing in the present study, and next-generation sequencing studies from China and USA (TCGA). (**C**) Mutations in the GPCR-mediated PI3K pathway. Bar indicates the combined frequency of mutation in the GPCR-mediated PI3K pathway in DTCs of the present study and other studies. (**D**) Interactome shows the interacting network of LPAR4 protein. The network nodes represent proteins and the red-colored node indicates the query protein and first shell of interactors. Bond represents protein-protein associations. Bond color and the type of associations are as mentioned above in Figure 3D. (**E**) Schematic diagram of the GPCR (LPAR4)-mediated PI3K signaling pathway. Growth factor activates the LPAR4 and activated LPAR4 triggers potential downstream signaling members such as PIK3CA, PTEN, and AKT, and the active PI3K pathway leads to growth, cell survival and inhibition of apoptosis.

As we found a low frequency of mutations in the GPCR-mediated PI3K pathway in the pediatric and adult DTCs of Saudi Arabian origin, we attempted to analyze the TCGA data of DTCs to determine whether the different cohort analyzed in the TCGA show a different prevalence of mutations in this pathway. As seen in Figure 4A OncoPrint, 0.2% (1/507) of cases had a somatic missense mutation in *LPAR4*; 0.4% (2/507) of the cases had missense mutations in *PIK3CA* and 1.2 % (6/507) cases harbored *PTEN* mutations consisting mainly of missense mutations and deep deletions. Overall, the GPCR-mediated pathway genes showed mutations in only 1.8% (9/507) of PTCs (Figure 4A–4C). This reflects low prevalence of mutations in this pathway regardless of the ethnic population and age group (pediatrics and adults) (Figure 4B–4E; Table 3).

**Table 3:** Mutations of GPCR-mediated PI3K pathway genes in differentiated thyroid cancer of various ethnic populations

Genes	Saudi Arabia (Sanger sequencing)^*^	China (Whole Exome Seq.)^1^	United States of America, TCGA (Whole Genome seq.)^2^
	[Mutations/Samples (Percentage)]	[Mutations/Samples (Percentage)]	[Mutations/Samples (Percentage)]
*LPAR4*	0/293 (0%)	4/91 (4.4%)	1/507 (0.2%)
*PIK3CA*	5/293 (1.7%)	1/91 (1.1%)	2/507 (0.4%)
*PTEN*	3/293 (1%)	1/91 (1.1%)	6/507 (1.18%)
Overall	8/293 (2.7%)	6/91 (6.6%)	9/507 (1.8%)

^*^Current study; ^1^Pan *et al*. 2016; ^2^Agrawal *et al*. 2014.

### TCGA data analyses of PDTCs and ATCs (aggressive thyroid cancers) show a relatively high incidence of mutations in GPCR-mediated PI3K pathway genes

**Figure 5:**
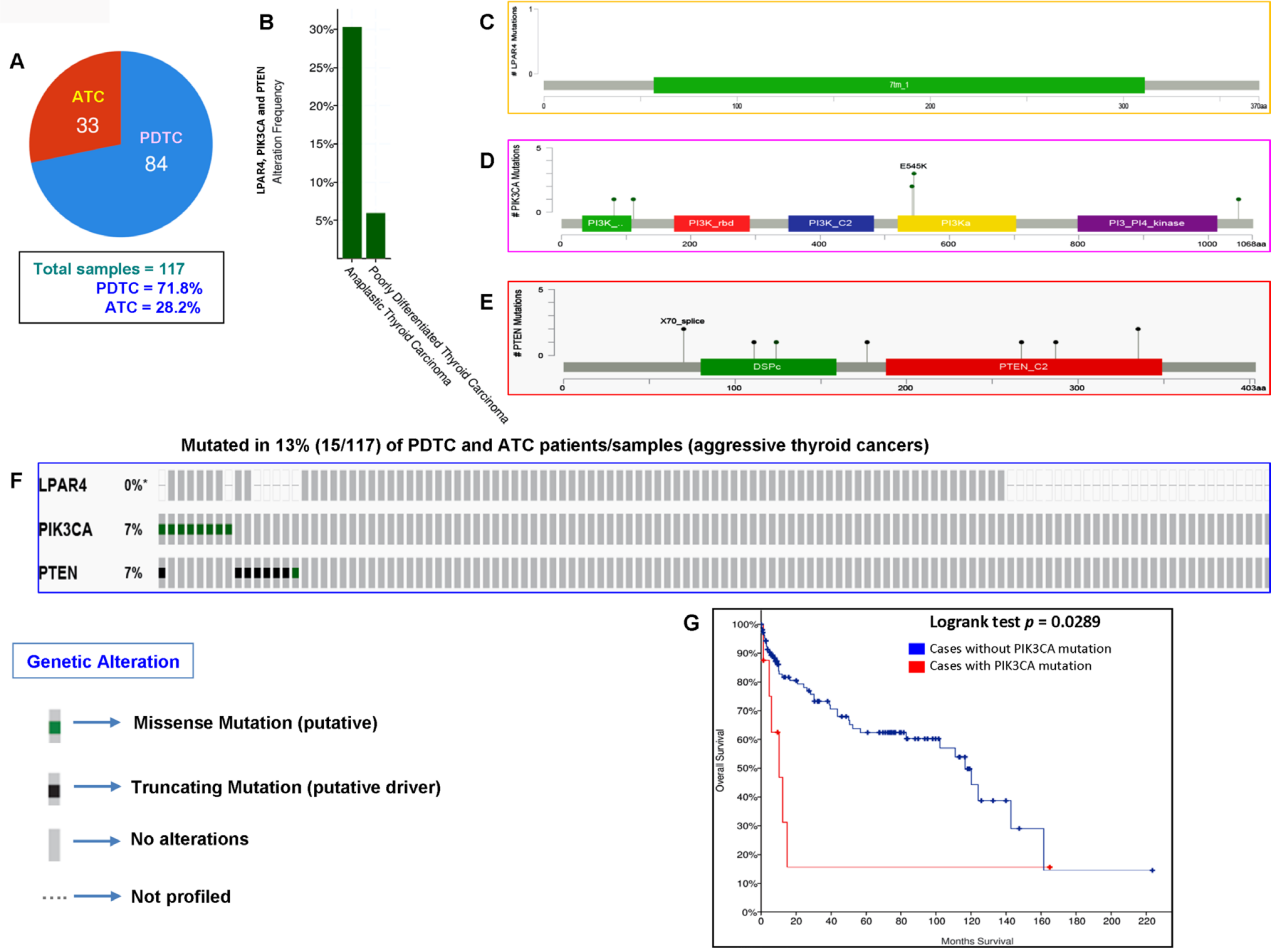
Prevalence of GPCR-mediated PI3K pathway mutations in PDTC and ATC (aggressive thyroid cancers). (**A**) Pie chart of aggressive thyroid cancers (PDTC and ATC). The chart shows the type of tumors, the number of samples in each type and total number of samples studied. (**B**) The histogram indicates the mutation frequency of GPCR-mediated PI3K pathway genes in thyroid cancers. The bar shows the overall frequency of *LPAR4*, *PIK3CA* and *PTEN* mutations in 6% and 30% of PDTC and ATC patients, respectively. (**C**–**E**) Mutation tab. The schematic diagrams indicate GPCR-mediated PI3K members (from top) LPAR4, PIK3CA (p110α) and PTEN protein domains and position of a particular mutation. The length of the line connected between mutation annotation and protein directly correspond to the number of mutated samples. The most frequent mutation is indicated in the diagram. (**F**) The OncoPrint tab. Tab shows the *LPAR4, PIK3CA* and *PTEN* mutations across the PDTC and ATC (MSKCC cohort). Each row represents a particular gene of GPCR-mediated PI3K pathway and each column shows a tumor sample. The green squares plotted on the columns are non-synonymous mutations. (**G**) Survival curve. The diagram is the Kaplan–Meier plot of overall survival of aggressive thyroid cancer-bearing patients (PDTC and ATC) absence or presence of *PIK3CA* mutations in blue and red color, respectively.

In our current study, analysis of the GPCR-mediated PI3K pathway in the aggressive phenotypes of thyroid cancer (PDTC and ATC) showed no mutation. However, we had a limited number of cases with these aggressive forms (13 cases only). We, therefore, analyzed the TCGA data of PDTCs and ATCs to determine whether the results we obtained corroborates with the TCGA data of the different ethnic populations. As illustrated in Figure 5A and 5B, this pathway harbors mutations in 30% (10/33) and 6% (5/84) of ATC and PDTC, respectively. As seen in Figure 5C–5F, each *PIK3CA* and *PTEN* genes harbored somatic mutations in 7% (8/117) of aggressive thyroid cancers (PDTCs and ATCs) while no mutation was found in *LPAR4* gene. Overall the GPCR-mediated pathway genes mutations were detected in 13% (15/117) of aggressive thyroid cancers (PDTCs and ATCs). These results suggest that the mutational prevalence of this pathway gene may be different among various ethnic cohorts. Furthermore, our analyses of this pathway genes revealed that the presence of *PIK3CA* mutation significantly predicts a poor overall survival (*p* = 0.0289) in patients with aggressive phenotypes suggesting a vital role of *PIK3CA* mutation in PDTCs and ATCs (Figure 5G).

### Construction of mutational landscape and mutated genes-mediated interactome reveals a complex multi-protein interaction network in aggressive thyroid cancers (PDTC and ATC)

**Figure 6:**
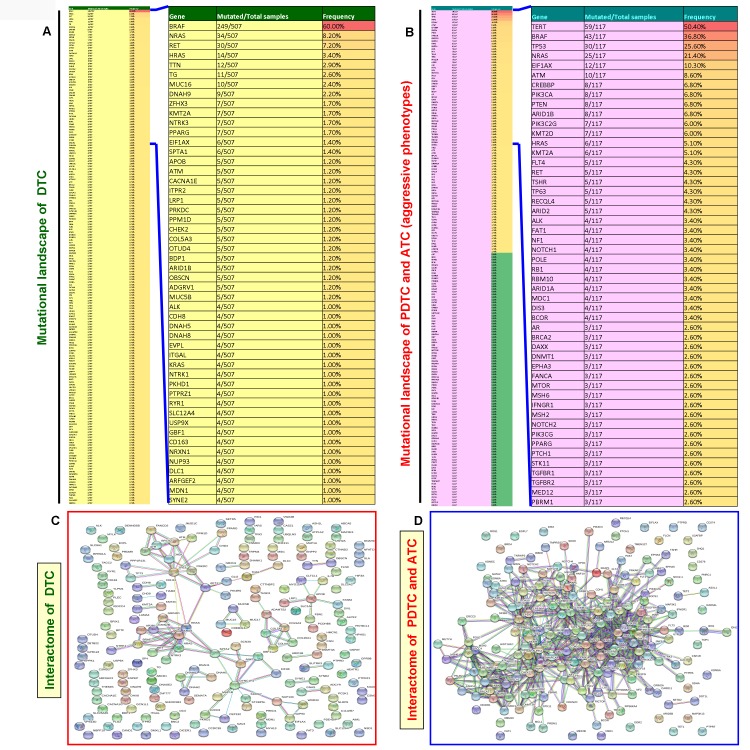
Mutated gene-mediated interactome of DTC and aggressive thyroid cancers (PDTC and ATC). (**A**) Mutational landscapes of DTC. The thin column shows the top 188 mutated genes in DTC and extracted after analyses of TCGA data of DTC with a gene mutational prevalence cutoff of ≥ 0.70% in cBioPortal. The projected wide column indicates the top 50 mutated genes in DTC. (**B**) Mutational landscapes of aggressive thyroid cancers (PDTC and ATC). The thin column shows 186 genes which include all the mutated genes in PDTC and ATC. These genes were extracted as explained in Figure 6A. The projected wide column indicates the top 50 mutated genes in aggressive thyroid cancers (PDTC and ATC). (**C** and **D**) The interaction network (**C** and **D**) show the mutated gene-mediated interactome of DTC and aggressive thyroid cancers (PDTC and ATC), respectively. The interactome of DTC and aggressive thyroid cancers (PDTC and ATC) was constructed by STRING v10 after extracting the full set of mutated genes indicated in the mutational landscape in (**A** and **B**), respectively.

Unlike DTCs, the PDTCs and ATCs are rare but aggressive thyroid cancers [7, 8]. Therefore, to find whether there is any difference in the mutational landscape and mutated genes-mediated protein-protein interaction network pattern between DTC and aggressive thyroid cancer (PDTC and ATC), we constructed and analyzed the mutational landscape and mutated genes-mediated interactome for DTC and aggressive thyroid cancers. In the mutational landscape, our analysis of TCGA data of DTCs showed 3600 mutated genes. We found 186 mutated genes in the TCGA data of aggressive thyroid cancers (PDTC and ATC). As illustrated in Figure 6A and 6B, we used the top 188 mutated genes for building the mutational landscape and protein-protein interaction network that covered all the mutated genes with a mutational prevalence of ≥ 0.70% in DTC and ≥ 0.80% in aggressive thyroid cancers. Projection of a top 50 mutated genes in DTC and aggressive thyroid cancers capped all the mutated genes with a mutational prevalence of ≥1% and 2.6%, respectively. Interestingly, compared with the DTC-specific interactome (3 major clusters include BRAF, NRAS, and P53), the aggressive thyroid cancers-mediated interactome reveals very complex (>13 major clusters by vital protein molecules such as NRAS, P53, AKT3, BRAF, TERT, PTEN, PIK3CA, EGFR, RAC1, CTNNB1, EBP300, CREBBP, MAPK1, etc.) and multi-node interactions in each protein molecule. Overall, it also shows the involvement of more number of mutated protein molecules and formation of multiple highly clustered interaction networks (Figure 6C and 6D. This interaction network truly reflects the complexity and advanced disease pattern of aggressive thyroid cancer phenotypes, PDTC and ATC.

### Analyses of pan-cancer cell lines in TCGA reveal highly frequent mutations of GPCR-mediated PI3K pathway genes

**Figure 7:**
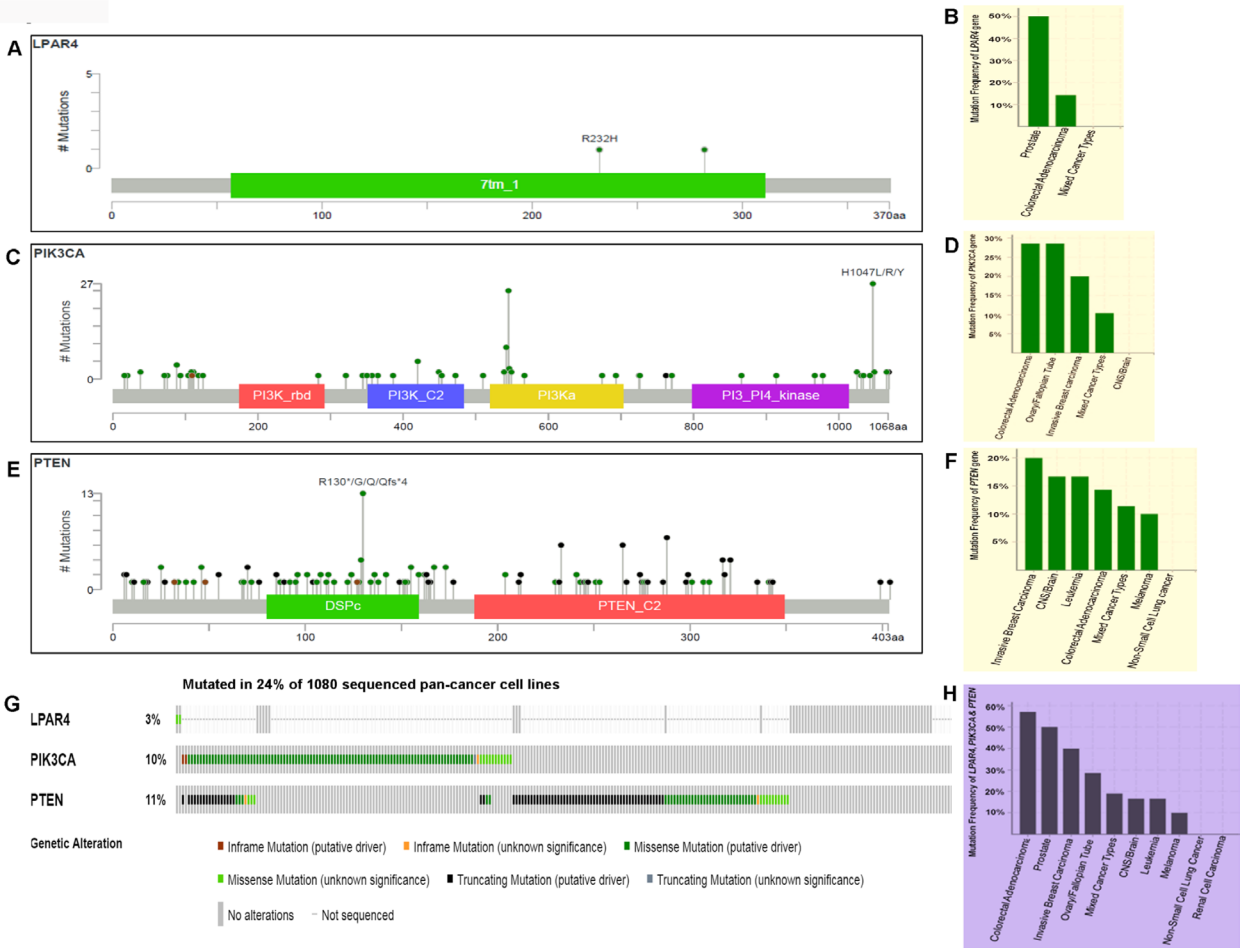
Prevalence of somatic mutations in genes of GPCR-mediated PI3K pathway in the pan-human cancer cell lines. (**A**) Schematic diagram of LPAR4 protein. The diagram shows the domains and the positions of somatic mutations. The length of the line connecting the indicated mutation to the protein represents the number of samples which is positive for the mutation. The labeled mutation in the diagram represents the most recurrent mutation. (**B**) The frequency of LPAR4 mutation. Bars indicate the frequency of *LPAR4* gene mutations across the human cancer cell lines. Only positive and a few negative cases are shown in the figure. (**C**) Schematic diagram of p110α protein (PIK3CA). The diagram shows various domains and the positions of somatic mutations as stated above in Figure 7A. (**D**) The frequency of PIK3CA (p110α) mutation. Bars indicate the frequency of somatic mutations of the *PIK3CA* gene across the human cancer cell lines as explained in Figure 7B. (**E**) Schematic diagram of PTEN protein. The diagram shows the domains and the positions of somatic mutations as stated above in Figure 7A. (**F**) The frequency of PTEN mutation. Bars indicate the frequency of somatic mutations of the PTEN gene across the human cancer cell lines as explained in Figure 7B. (**G**) OncoPrint of GPCR-mediated PI3K pathway of pan-human cancer cell lines. The OncoPrint bar summarizes genomic alterations in *LPAR4, PIK3CA* and *PTEN* genes across the TCGA sample set of 1080 human pan-cancer cell lines. Each horizontal row indicates a gene and each vertical column shows a tumor sample. Green bars show nonsynonymous mutations, blue bars show homozygous deletions, black shows truncating mutations, no color indicates the absence of mutation and gap shows not sequenced. (**H**) Frequency of GPCR-mediated PI3K pathway genes (combined) across the type of human cancer cell lines. Highest and the lowest frequency of these pathway mutations were observed in colorectal adenocarcinoma and melanoma, respectively.

To assess the rates of GPCR-mediated PI3K pathway and whether mutations in this pathway play role in other human cancers, we initially analyzed the pan-cancer cell lines sequenced data from TCGA. From the OncoPrint (Figure 7A–7F), 3.3% (2/60) of cases had a somatic mutation in *LPAR4* gene and all the alterations were missense mutations. A high rate of 50% (1/2) *LPAR4* mutations was found in prostate cancer cell line but only two cell lines were reported. This was followed by colorectal cancer cell lines [14.29% (1/7)]. Overall, 10.3% (111/1080) of cell lines had somatic mutations in the *PIK3CA* gene. Colon and ovarian cancer cell lines had a high frequency of the *PIK3CA* mutation of 28.57% (2/7) followed by invasive breast carcinoma [20% (1/5)]. Overall, 11.2% (121/1080) cell lines had somatic mutations in the *PTEN* gene. The *PTEN* showed mutations in 20% (1/5) of breast cancer, 16.67% (1/6) of brain and leukemia followed by 14.29% (1/7) of colon cancer cell lines. Overall this pathway contributed to 24.8% of cases of pan-cancer cell lines (Figure 7G). As shown in Figure 7H, this pathway genes had frequent mutations [57.14% (4/7)] in colon cancer cell lines followed by 50% (2/4), 40% (2/5), 28.57% (2/7) of prostate, breast, ovarian cancer cell lines, respectively. These results suggest that the pathway may be frequently mutated in solid tumors.

### TCGA data analyses display frequent mutations of GPCR-mediated PI3K pathway genes in human solid cancers

**Figure 8:**
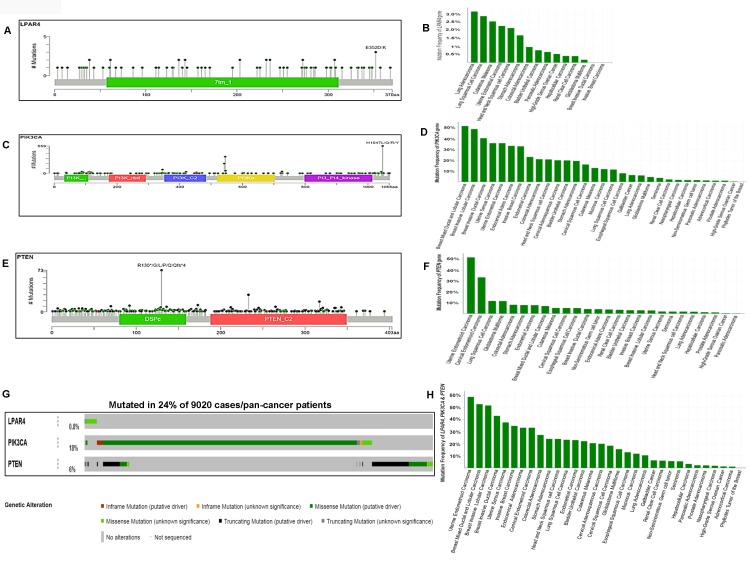
Somatic mutations of the GPCR-mediated PI3K pathway in pan-human cancers (solid tumors) and their association with patient survival. (**A**) Schematic diagram of LPAR4 protein. The diagram shows the domain and the positions of somatic mutations. The length of the line connecting the indicated mutation to the protein represents the number of samples which is positive for the mutation. The labeled mutation in the diagram represents the most recurrent mutation. (**B**) The frequency of LPAR4 mutation in pan-human cancers (solid). The histogram shows the somatic mutation frequencies of the *LPAR4* gene mutations across cross cancer studies. (**C**) Schematic diagram of p110α protein (PIK3CA). The diagram shows various domains and the positions of somatic mutations identified in pan-human cancer (solid) as stated above in Figure 8A. (**D**) The frequency of PIK3CA mutation in pan-human cancers (solid). The histogram shows the somatic mutation frequencies of the *PIK3CA* gene cancer studies. (**E**) Schematic diagram of PTEN protein. The diagram shows various domains and the positions of somatic mutations identified in pan-human cancer (solid) as stated above in Figure 8A. (**F**) The frequency of PTEN mutation in pan-human cancers (solid). The histogram shows the somatic mutation frequencies of the PTEN gene across cancer studies. (**G**) OncoPrint of GPCR-mediated PI3K pathway of pan-human cancer. The OncoPrint bar summarizes genomic alterations in *LPAR4, PIK3CA* and *PTEN* genes across the TCGA sample set of 9020 pan-cancer samples. Each horizontal row indicates a gene and each vertical column shows a tumor sample. Green bars show nonsynonymous mutations, blue bars show homozygous deletions, black shows truncating mutations, no color indicates the absence of mutation and gap shows not sequenced. (**H**) Frequency of GPCR-mediated PI3K pathway gene mutations (combined) across the type of pan-human cancer. The highest frequency of these pathway mutations was observed in uterine endometrioid carcinoma and various types of breast carcinoma.

To confirm the high frequency of GPCR-mediated pathway mutations found in human cancer cell lines, we analyzed the pan-cancer sequencing data from TCGA. As seen in Figure 8A and 8G, *LPAR4* showed 0.8% (75/9020) mutation in pan-human solid cancers. Lung adenocarcinoma revealed 3.18% (21/660), the highest frequency of mutation in this gene followed by 2.89% (14/484) in lung squamous cell carcinoma and 2.56% (1/39) cutaneous melanoma (Figure 8B). *PIK3CA* had mutations in 18% (1625/9020) of tumors (Figure 8C and 8G) and it was frequently mutated in breast mixed ductal and lobular carcinoma 51.72% (45/87) followed by 48.94% of breast invasive lobular (69/141) and 40.47 % of ductal carcinoma (607/1500), respectively Figure 8D. *PTEN* showed a 6% (551/9020) of mutations in pan-human solid cancers (Figure 8E and 8G). As illustrated in Figure 8F, *PTEN* harbored high frequent mutations in uterine endometrioid carcinoma [51.79% (159/307)] next to cervical endometrioid carcinoma [33.33% (1/3)] and lung squamous cell carcinoma 11.57% (56/484)]. Overall this gene set was somatically mutated in 24.8% (Figure 8G) of solid cancers. This pathway’s mutations were frequently observed in 58.96% (181/307) of uterine endometrioid carcinoma, 52.87% (46/87) of breast mixed ductal and lobular carcinoma, 51.77% (73/141) of breast invasive lobular carcinoma (Figure 8H). These results show that this pathway is frequently mutated in human pan-tumor samples and could be a potentially important therapeutic target.

### GPCR-mediated PI3K pathway genes significantly co-occur in human solid cancers

To determine any relationship between oncogenic activation and loss of tumor suppressor function in the GPCR-mediated PI3K pathway, mutations in the *LPAR4*, *PIK3CA,* and *PTEN* mutations were analyzed in relation to each other both in pan-cancer cell lines and pan-solid tumor samples (Table 4). Our analyses demonstrated that *PIK3CA* and *PTEN* mutations co-occurred significantly in pan-cancer cell lines (*p* = <0.001). However, *LPAR4* vs *PIK3CA* and *LPAR4* vs *PTEN* were not related in either mutually exclusive or a co-occurring manner (*p* = 0.805 and *p* = 0.788, respectively) in pan-cancer cell lines. To see the consistency of this relationship, we also analyzed the co-occurrence of mutations in these genes in pan-tumor cases. This showed that *PIK3CA* and *PTEN* mutations significantly co-occurred (*p* = <0.001). In addition, *LPAR4* and *PTEN* also exhibited a significant co-occurrence (*p* = 0.003). However, *LPAR4* and *PIK3CA* did not show any significant co-occurrence and/or mutual exclusivity (*p* = 0.499). These results suggest a significantly combined role of *PIK3CA* with *PTEN* and *LPAR4* with *PTEN* in the human solid tumors. We also performed individual network analysis and found that *PIK3CA* and *PTEN* could strongly interact and this was consistent with our data as it co-occurred significantly in pan-cancer cell lines and tumors. *LPAR4* could interact strongly with various PI3K pathway genes including *PIK3CA* except for *PTEN* (Figure 4D).

**Table 4:** Co-occurrence and mutual exclusivity of somatic mutations in GPCR-mediated PI3K pathway genes in pan-cancer

Samples	Gene A	Gene B	Neither	A Not B	B Not A	Both	Log Odds Ratio	*p*-Value	Tendency	Significance
Cancer cell lines	*PIK3CA*	*PTEN*	876	83	93	28	1.156	**<0.001**	Co-occurrence	**Significant**
	*LPAR4*	*PIK3CA*	967	2	111	0	<-3	0.805	Mutual exclusivity	No
	*LPAR4*	*PTEN*	957	2	121	0	<-3	0.788	Mutual exclusivity	No
Pan-cancers	*PIK3CA*	*PTEN*	7058	1452	359	171	0.853	**<0.001**	Co-occurrence	**Significant**
	*LPAR4*	*PTEN*	8430	60	519	11	1.091	**0.003**	Co-occurrence	**Significant**
	*LPAR4*	*PIK3CA*	7358	59	1591	12	–0.061	0.499	Mutual exclusivity	No

## DISCUSSION

PI3K pathway genes are more frequently altered in high-grade thyroid cancers particularly in FTCs, PDTCs, and ATCs than in the well-differentiated types. [5, 16, 7, 8, 21–23]. Previous studies focused mostly on *PIK3CA* and/or *PTEN* of the PI3K pathway while several other studies examined individual genes of the PI3K pathway in thyroid cancers mainly FTCs and ATCs but have not studied the complete axis [27, 14, 15, 28]. Recently, WES of a Chinese population with PTCs revealed frequent mutations in the GPCR-mediated PI3K pathway and identified mutations of *LPAR4* as novel PTC driver alterations [26]. LPAR4 is implicated in cell proliferation, migration, invasion, and tumorigenesis [24, 25]. However, to date, knowledge on the somatic mutations of *LPAR4* in DTCs remains unknown except in the Chinese population [26]. *LPAR4* mutations were not studied in pure pediatric and adult DTC groups, and aggressive thyroid cancers (PDTC and ATC). Furthermore, the prevalence of genetic alterations of this axis has not been explored in DTC, PDTC, and ATC from Saudi Arabia, a population of unique genetic makeup with high rates of thyroid cancer and consanguinity. We therefore specifically studied genetic alterations of the *LPAR4*-*PIK3CA*-*PTEN* of the PI3K pathway axis in pediatric and adult DTCs, PDTCs and ATCs derived from Saudi population. To determine if there is any difference in the rates of these mutations between different ethnic populations, we also analyzed this axis in the TCGA data of PTCs and aggressive thyroid cancers (PDTC and ATC). We also analyzed this axis in human pan-cancer cell lines and other solid tumors to understand the relative significance of the PI3K pathway in human tumorigenesis.

Our study revealed GPCR-mediated PI3K pathway mutations in 3.37% and 2.45% of pediatric and adult DTCs, respectively. In addition, pediatric and adult DTCs showed no significant differences in the prevalence of these mutations. Similarly, low frequent (4.8%) mutations have also been found in this axis (3.6%, *PIK3CA* and 1.2%, *PTEN*) in DTC from a Greek population [29]. However, a relatively high frequency (6.6%) of this pathway-mutations was found in a Chinese population with DTC [26] suggesting that ethnic differences, sample selection, and sequencing method are likely to influence the prevalence of mutations in this pathway. Moreover, consistent with previous studies [28–30], our study also suggests that PI3K pathway alterations are quite rare events and may indicate that the GPCR-mediated PI3K pathway genes are mutated only in a small fraction of DTCs. Although somatic mutations of *PIK3CA* and *PTEN* genes have previously been documented in sporadic thyroid cancers, they were mostly observed in high-grade tumors of FTCs and ATCs [31, 14, 30, 16].

In this study, of 293 DTC cases, 5 harbored 7 different *PIK3CA* mutations as two cases had concomitant mutations (Q530R; A533V and S553T; T1659del). Out of the 7 *PIK3CA* mutations, 5 and 2 were identified in the helical and kinase domain, respectively. Most of the mutations identified were localized in non-hotspot codons of the helical and kinase domains with the exception of a helical domain mutation (E545Q). Further, we identified *PTEN* mutations in 3 of 293 cases. All the *PIK3CA* and *PTEN* mutations identified in this study were localized to various functional domains. PI3K pathway mutations are reported to be mutually exclusive in human cancers including thyroid cancer. However, only one case had concomitant *PIK3CA* and *PTEN* mutation suggesting a weaker oncogenic potential of *PIK3CA* and requirement of a loss of tumor suppressor-like *PTEN*. Recent deep sequencing of DTCs (PTCs) identified *PIK3CA* and *PTEN* mutations in samples with distant metastasis [32] suggesting that the DTCs with these mutations are likely to progress into aggressive poorly differentiated thyroid cancer (PDTCs). Further, *PIK3CA*/*PTEN* mutation-positive samples were negative for the most commonly identified *TERT* promoter mutations. Nonetheless, of the 5 *PIK3CA* mutation positive cases, 2 had concomitant *BRAF* mutation. *BRAF* mutation in combination with a gain-of-function *PIK3CA* or loss-of-function *PTEN/TP 53* mutation scenario has only been seen in the ATCs. A recent study demonstrated that *BRAF* mutant induced PTC was indolent and could not lead to end-stage disease. Similarly, *PIK3CA* mutant was unable to transform thyrocytes on its own but when co-expressed with *BRAF* mutant, that lead to the development of lethal ATC in mice suggesting that tumors bearing both *BRAF* and *PIK3CA* mutations are likely to progress into PDTCs and lethal ATCs [33]. On the other hand, thyroid cancer patients with *PIK3CA* mutations were shown to have a distinct pathological profile as *PIK3CA* mutations are likely to arise during the tumor progression [34]. Furthermore, 2 of 3 *PTEN* mutation-positive cases had *BRAF* mutation. It has been shown that BRAF^V600E^ and PTEN loss facilitates the progression of thyroid cancers when the fibroblast-mediated collagen remodeling takes place within the tumor microenvironment suggesting a possibility of synergism in tumor progression [35].

In addition, we analyzed TCGA, a large series of DTCs (n=507) from a completely different ethnic background [4]. Consistent with our data, we observed a low prevalence (1.8%) of GPCR-mediated PI3K pathway gene mutations (0.2% of *LPAR4*, 0.4% of *PIK3CA* and 1.18% of *PTEN*). The prevalence of both our study (2.7%) and TCGA (1.8%) is relatively lower compared to that reported from the study from Chinese cases (6.6%) (Table 3) [26]. Although the low prevalence of GPCR-mediated PI3K pathway mutations is identified both in this study and TCGA data, they are located in the important functional domains including helical and kinase domains and interact with the important key players in the signaling network, and hence the mutations are likely to be oncogenic/tumor progression drivers.

The PDTCs and ATCs are rare but aggressive phenotypes of thyroid cancers particularly the ATC which is deadly cancer with less than 4 months median survival from the initial diagnosis [7, 8]. Analyses of these aggressive thyroid cancers in our study revealed no mutation in the GPCR-mediated PI3K pathway (*LPAR4*-*PIK3CA*-*PTEN*) genes. In contrast, analyses of TCGA data of aggressive thyroid cancers (PDTC and ATC) showed an overall 13% mutation rate in this pathway. Interestingly, this pathway had mutations in 30% of ATCs but only in 6% of PDTC. *PIK3CA* was found to be mutated in 7% of cases and *PTEN* mutations were also found in 7% while mutation was not detected in *LPAR4*. To assess whether these relatively frequent mutations have any prognostic value, we performed survival analysis in the genes of GPCR-mediated PI3K pathway in aggressive thyroid cancer (PDTC and ATC). Patients bearing *PIK3CA* mutations showed a significant poor overall survival (*p* = 0.0289) compared with people bearing wild-type *PIK3CA* suggesting the important role of this gene in aggressive thyroid cancer. Although the PI3K pathway is frequently genetically altered in PDTC and ATC [31, 14, 30, 16], the sample size is limited in our study and that could explain the absence of mutations in this pathway in the 13 cases of PDTC and ATC we studied. Alternatively, ethnic differences might be the reason for this apparent difference in the rate of mutations in PDTC and ATC in our study compared to TCGA data. Moreover, construction of mutational landscape and mutated gene-derived protein-protein interaction network (interactome) prominently reflected the simple disease pattern of DTC and complexity of the advanced disease (PDTC and ATC) and may assist in guiding the right molecular therapeutic target.

As GPCR-mediated PI3K pathway axis has never been analyzed in other solid malignancies, we mined pan-cancer cell lines (*n =* 1080) and tumor (*n =* 9020) from TCGA data. Unlike the low prevalence observed in DTCs and thyroid TCGA, both pan-cancer cell lines (24.8%) and tumors (24.8%) had high mutation frequency which is even higher than (6.6%) that reported in DTC from a Chinese population [26]. *LPAR4* mutations in pan-tumors (0.8%) were comparable to that of DTCs though pan-cancer cell lines had a slightly higher prevalence (3.3%). *LPAR4* mutations have previously been reported in 16% (1/6) only in human colon cancer cell lines [36] and recently in PTCs [26]. However, *PIK3CA* and *PTEN* showed more frequent mutation rates in pan-cancer cell lines and tumors and were very consistent with the previous reports [37, 38, 16] suggesting that more proven contribution of this pathway gene in the pathogenesis of solid pan-tumors.

Co-occurrence and mutual exclusivity inferred by a statistical analysis could show a rough relationship between different genes and they could serve as a platform for drug discovery. We, therefore, analyzed *LPAR4*, *PIK3CA* and *PTEN* genes in TCGA data of pan-cancer cell lines and tumors. This showed that *PIK3CA* and *PTEN* could significantly co-occur in pan-cancer cell lines (*p* = <0.001) and tumors (*p* = <0.001). As the interactome could reveal the interaction among the different genes, we performed network analysis and found that *PIK3CA* and *PTEN* could strongly interact and this was consistent with our data as it co-occurred significantly in pan-cancer cell lines and tumors. It has been widely demonstrated that *PIK3CA* and *PTEN* mutations were mutually exclusive; suggesting that tumorigenic signaling through the PI3K pathway could occur either with activation of *PIK3CA*/inactivation of *PTEN* [38, 39]. In contrast, it has been reported that coexistence of *PIK3CA/PTEN* mutations at high frequency (26%) in endometrial carcinoma. Further, *PIK3CA* mutations were more frequent in tumors with *PTEN* mutations (46%) compared with those without *PTEN* mutations (24%) [40] and this concomitant mutations (1.3%) have also been reported from Chinese breast cancers [41]. Further, an oncogenic *PIK3CA* mutation coupled with *PTEN* loss was shown to initiate ovarian tumors in mice [42]. This unique genetic representation indicates that the combination of *PIK3CA/PTEN* alterations might play an important synergistic role in tumorigenesis of these cancers. Moreover, *LPAR4* and *PTEN* could co-occur in pan-tumor samples (*p* = 0.003). Nevertheless, network analysis revealed that *LPAR4* could interact strongly with various PI3K pathway genes including *PIK3CA* except for *PTEN*. In contrast, *LPAR4* was rarely analyzed for mutation except for a report from Chinese PTCs [26]. We did not find any significant mutual exclusivity among *LPAR4*, *PIK3CA,* and *PTEN*. Though data mining studies are more informative, they cannot draw a definite conclusion on the initiating event for the malignancy and only the prospective experimental studies could confirm that hypothesis.

In conclusion, data from our study and TCGA data of thyroid showed a similar low prevalence of mutations in GPCR-mediated PI3K pathway genes. Pediatric and adult cases showed no significant differences in mutational rates in this pathway. These results suggest that this pathway contributes to the pathogenesis of a smaller portion of DTCs but may have a major role in the progression of tumors harboring mutations in this pathway, leading to more aggressiveness and metastasis leading to PDTC and ATC. In addition, TCGA data analyses reveal that GPCR-mediated PI3K pathway genes are frequently mutated and they are likely playing a key role in the pathogenesis of PDTC and ATC and other human solid malignancies.

## MATERIALS AND METHODS

### Tumor tissue samples and their histopathological features

Tumor samples were collected from the Department of Pathology, King Faisal Specialist Hospital and Research Centre (KFSH&RC), Riyadh, and the study was approved by the Institutional Review Board (IRB) (RAC-2130015). We used a total of 323 samples which includes 17 multinodular goiters (MNGs), 89 pediatric, 204 adult DTCs and 13 aggressive thyroid cancers. Pediatric DTCs includes 72 (81%) conventional papillary thyroid cancer (CPTC), 7 (7.9%) follicular variant-papillary thyroid cancer (FV-PTC), 2 (2.2%) tall cell variant PTC (TPTC), 3 (3.4%) diffuse sclerosing type papillary thyroid cancer (DSC), 3 (3.4%) follicular thyroid cancer (FTC) and 2 (2.2%) Hürthle Cell Cancer (HCC). The pediatric DTCs include all patients with the age at diagnosis of ≤ 18 years. Adult DTCs includes 114 (55.9%) CPTC, 55 (27%) FV-PTC, 29 (14.2%) TPTC, 3 (1.5%) HCC and 3 (1.5%) DSC. All the aggressive thyroid cancers were adults and consist of 10 (77%) poorly differentiated thyroid cancer (PDTC) and 3 (23%) anaplastic thyroid cancer (ATC).

### Genomic DNA extraction

Slices of ~10-micron thickness of tumor samples were dissected from formalin fixed and paraffin embedded tissue (FFPE) after histological diagnosis was confirmed by an experienced endocrine pathologist (H.A). Genomic DNA was extracted as previously described [43] using the Gentra Puregene DNA extraction kit (Qiagen, Valencia, CA, USA).

### PCR amplification and sequencing

We PCR amplified exon 1 of the *LPAR4* gene using a forward primer with a 5’ M13 tag (LPAR4FW- 5’-GTA AAA CGA CGG CCA GTC TCT TCG CAA GCC TGC TAC-3’and a reverse primer with a 5’ M13 tag (LPAR4RV-5’-CAG GAA ACA GCT ATG ACC GCA AGG CAC AAG GTG ATT GG-3’ and the PCR conditions were as follows: initial denaturation at 94º C for 2 min and 35 cycles consisting denaturation at 94º C for 30 sec, annealing at 60º C for 30 sec with extension at 72º C for 45 sec followed by a 7 min final extension at 72º C. Primers for exons 9 & 20 of *PIK3CA* gene and exons 5, 6, 7 and 8 of *PTEN* gene was as reported previously [14]. All the mutation-positive cases were further screened for the common mutations in exon 15 of *BRAF* and *TERT* promoter using primers as described earlier [10, 18]. We limited our search to these exons as mutations were mostly harbored in these exons. The PCR amplicons were confirmed on 2.5% agarose gel and the successfully-amplified samples were subjected to direct sequencing using the Big Dye Terminator v3.1 cycle sequencing kit and by ABI PRISM 3730X1 genetic analyzer (Applied Biosystems). Identified mutations were confirmed in both forward and reverse directions by an independent PCR amplification and sequencing. The sequencing results were read against the GeneBank Accession No: *LPAR4* (NM_005296.2)*, PIK3CA* (NM_006218.3), *PTEN* (NM_000314.6)*, BRAF* (NM_004333.4) and *TERT* (AF098956.1).

### 3D structure

The p110α protein in complex with p85 (niSH2) was downloaded from https://www.rcsb.org/ using the PDB ID: 2RDO. Amino acid residues of p110α (*PIK3CA*) somatic mutations identified in pediatric/adult DTCs were plotted in p110α native protein structure using SWISS PDB viewer as indicated previously [35]. The structure of PTEN was downloaded using the PDB ID: 1D5R as mentioned above. *PTEN* mutations identified in pediatric/adult DTCs were viewed and plotted using NGL viewer, a WebGL based 3D viewer powered by MMTF (Macromolecular Transmission Format) as described before [44].

### Construction of interactive signaling network

Protein interacting network (interactome) for *LPAR4*, *PIK3CA* and *PTEN* protein molecules were constructed using a pre-computed database for the exploration and analysis of protein-protein interactions named STRING v10 (Search Tool for the Retrieval of Interacting Genes/Proteins) database [45]. All the protein-protein interaction networks were built with an interaction score of the highest confidence (0.900). The minimum required interaction score is 0.150, i.e., considered as low confidence.

### Construction of mutational landscape and mutated gene-mediated interactome

The mutational landscape was constructed by analyzing the respective DTC (TCGA) and aggressive thyroid cancers (PDTC and ATC) from the TCGA data (MSKCC) of the respective phenotype in cBioPortal. Mutated gene-based interactome (protein-protein interaction network) was built by STRING v10 [45]. Initially, the data was filtered and used only the genetically altered genes (only the genes with mutation and deletion) in related phenotype and all the genes with copy number variations (CNV) were excluded from our further analyses. Our analyses showed 3600 mutated genes in DTC. We therefore filtered the mutated genes using gene with ≥ 0.70% of mutation as a cutoff and that extracted 188 genes and used all these genes for constructing DTC interactome and out of these genes, top 50 were projected for better visibility. Similarly, in PDTCs and ATCs, we used the same cutoff of 188 which covered all the 186 mutated genes in PDTC and ATC and used them for constructing interactome and top 50 mutated genes were projected.

### TCGA data analyses for the mutation in the GPCR-mediated PI3K pathway (*LPAR4, PIK3CA,* and *PTEN*) genes

#### Differentiated thyroid cancer (DTC): TCGA data analyses

Data consisting of 507 DTCs from TCGA (Papillary Thyroid Carcinoma, TCGA, Cell 2014) were analyzed for this study and only mutations and deletions were included. Copy number variations (CNV) were excluded from this study.

#### Aggressive thyroid cancer: poorly-differentiated thyroid cancer (PDTC) and anaplastic thyroid cancer (ATC) - TCGA data analyses

TCGA data of 117 aggressive thyroid cancer samples that include 84 PDTCs and 33 ATCs (Poorly-Differentiated and Anaplastic Thyroid Cancers, MSKCC, JCI 2016) were analyzed in this study. Copy number variations (CNV) were excluded in this study while only the mutations and deletions were included.

#### Pan-human cancer cell line: TCGA data analyses

In total, 1080 pan-cancer cell lines from 2 different studies, Cancer Cell Line Encyclopedia (Novartis/Broad, Nature 2012) and NCI-60 Cell Lines (NCI, Cancer Res. 2012) were analyzed.

#### Pan-human solid cancer sample: TCGA data analyses

A total of 9020 pan-solid cancer samples (derived from 9017 patients) from 20 different studies were analyzed which include Adrenocortical carcinoma (TCGA, Provisional), Bladder Urothelial carcinoma (TCGA, Nature 2014), Breast Cancer (METABRIC, Nature 2012 & Nat Commun 2016), Cervical Squamous Cell carcinoma and Endocervical Adenocarcinoma (TCGA), Colorectal Adenocarcinoma (DFCI, cell Reports), Esophageal Squamous Cell Carcinoma (UCLA, Nat Genet 2014), Gallbladder Carcinoma (Shanghai, Nat Gene 2014), Genomic Hallmarks of Prostate adenocarcinoma (CPC-GENE, Nature 2017), Genomic Profile of Patients with Advanced germ Cell Tumors (MSK, JCO 2016), Glioblastoma (TCGA, Cell 2013), Head and Neck Squamous Cell carcinoma (TCGA, Nature 2015), Hepatocellular Carcinomas (Inserm, Nat Genet 2014), Kidney Renal Clear cell Carcinoma (TCGA, Nature 2013), Nasopharyngeal Carcinoma (Singapore, Nat Gene 2014), Ovarian Serous Cystadenocarcinoma (TCGA, Nature 2011), Pan-Lung Cancer (TCGA, Nat Genet 2016), Pancreatic Adenocarcinoma (QCMG, Nature 2016), Stomach Adenocarcinoma (TCGA, Nature 2014), Uterine Corpus Endometrial Carcinoma (TCGA, Nature 2013) and WES of pretreatment melanoma tumors (UCLA, Cell 2016) and excludes thyroid TCGA DTC data. All the data mentioned above were derived and various analyses were performed in the cBioPortal for Cancer Genomics (www.cbioportal.org).

### Statistical analyses

Mutual exclusivity/co-occurrence analysis of mutations in pan-cancer cell lines and pan-solid tumor samples were performed by calculating the odds ratio followed by Fisher’s exact test. Kaplan–Meier plots with a log-rank test were used to calculate the overall and disease-free survival of pan-solid tumors (except thyroid cancer) with at least one mutation or without mutation in the query gene. All the analyses were performed with the tools available in the cBioPortal for Cancer Genomics (www.cbioportal.org). A *p-*value < 0.05 was considered as statistically significant.
